# Association between the food and physical activity environment, obesity, and cardiovascular health across Maine counties

**DOI:** 10.1186/s12889-019-6684-6

**Published:** 2019-04-03

**Authors:** Allison C. Briggs, Adam W. Black, F. Lee Lucas, Andrea E. Siewers, Kathleen M. Fairfield

**Affiliations:** 1 Bowdoin College, Brunswick, ME, USA; 2 Center for Outcomes Research and Evaluation, Maine Medical Center Research Institute, 509 Forest Avenue, Suite 200, Portland, ME 04101 USA

**Keywords:** Built environment, Cardiovascular health, Health behaviors, Obesity

## Abstract

**Background:**

Accounting for nearly one-third of all deaths, cardiovascular disease is the leading cause of mortality and morbidity in the United States. Adverse health behaviors are major determinants of this high incidence of disease. Examining local food and physical activity environments and population characteristics in a poor, rural state may highlight underlying drivers of these behaviors. We aimed to identify demographic and environmental factors associated with both obesity and overall poor cardiovascular health (CVH) behaviors in Maine counties.

**Methods:**

Our cross-sectional study analyzed 40,398 Behavioral Risk Factor Surveillance System (BRFSS) 2011–2014 respondents alongside county-level United States Department of Agriculture (USDA) Food Environment Atlas 2010–2012 measures of the built environment (i.e., density of restaurants, convenience stores, grocery stores, and fitness facilities; food store access; and county income). Poor CVH score was defined as exhibiting greater than 5 out of the 7 risk factors defined by the American Heart Association (current smoking, physical inactivity, obesity, poor diet, hypertension, diabetes, and high cholesterol). Multivariable logistic regression models described the contributions of built environment variables to obesity and overall poor CVH score after adjustment for demographic controls.

**Results:**

Both demographic and environmental factors were associated with obesity and overall poor CVH. After adjustment for demographics (age, sex, personal income, and education), environmental characteristics most strongly associated with obesity included low full-service restaurant density (OR 1.34; 95% CI 1.24–1.45), low county median household income (OR 1.31; 95% CI 1.21–1.42) and high convenience store density (OR 1.21; 95% CI 1.12–1.32). The strongest predictors of overall poor CVH behaviors were low county median household income (OR 1.30; 95% CI 1.13–1.51), low full-service restaurant density (OR 1.38; 95% CI 1.19–1.59), and low fitness facility density (OR 1.27; 95% CI 1.11–1.46).

**Conclusions:**

In a rural state, both demographic and environmental factors predict overall poor CVH. These findings may help inform communities and policymakers of the impact of both social determinants of health and local environments on health outcomes.

**Electronic supplementary material:**

The online version of this article (10.1186/s12889-019-6684-6) contains supplementary material, which is available to authorized users.

## Background

Obesity is an important public health problem, affecting 39.8% of United States adults in 2015–2016 [1]. Maine has the highest adult obesity rate in New England, with this rate increasing from 28.2% in 2015 to 30% in 2016 [2]. Obesity is also a major risk factor for poor cardiovascular health (CVH), which is the leading cause of mortality and morbidity in the United States [3]. According to the American Heart Association (AHA), behavioral risk factors such as diet, physical activity, and smoking are the leading determinants of both obesity and poor CVH, with such modifiable behaviors accounting for 50% of cardiovascular deaths in the United States between 2009 and 2010 [4].

Rural states such as Maine are disproportionately burdened by poor CVH, in part because of high rates of poverty and behavioral risk factors [5]. Heart disease accounts for 20.8% of all deaths in Maine, and state obesity rates are higher than the national average [6]. Approximately 42% of Maine’s population lives in rural areas, with 11 out of 16 counties classified as rural [7]. These counties have higher poverty rates and lower annual incomes than urban Maine counties [5]. In Maine and other rural areas, residents tend to exhibit higher rates of adverse health behaviors, such as physical inactivity and poor diet, and are at greater risk of premature death and cardiovascular disease [8]. Identifying underlying drivers of these poor health behaviors could help reduce this burden of disease through targeted population health interventions.

A growing body of work has begun to examine the local built environment as a potential driver of health outcomes. The built environment encompasses aspects of the environment constructed by humans, such as transportation infrastructure, urban design, and man-made structures [9]. The food and physical activity environment refers to built environment components directly linked to diet and exercise, including food availability metrics (e.g., density of grocery stores), food access metrics (e.g., proximity of grocery stores), and recreation facility metrics [10]. Recent studies have begun to explore the relationship between the food and physical activity environment and behaviors such as diet [11] and physical activity [10] and health factors such as obesity [9], diabetes [12, 13], and cardiovascular disease [14, 15], though the studies have yielded mixed findings. Furthermore, environmental influences in rural areas may differ greatly from those in urban areas and may exhibit unexpected results [10]. A growing proportion of this work [12, 16] draws from census data compiled in the United States Department of Agriculture (USDA) Economic Research Service Food Environment Atlas, which assembles food environment indicators at the county, state, and regional level [7]. The Center for Disease Control’s (CDC) Behavioral Risk Factor Surveillance System (BRFSS) is a valuable source of population health data, gathering self-reported data on U.S. residents regarding health-related lifestyle behaviors, health conditions, and use of medical services [17].

We aimed to extend these analyses by examining the links between rural food and physical activity environments and the American Heart Association’s (AHA) new approach to CVH. In 2011, the AHA introduced a concept of “poor CVH score” that emphasizes the contributions of three lifestyle behaviors (smoking, poor diet, and physical inactivity) and four intermediate health factors (obesity, hypertension, diabetes, and high cholesterol) to CVH [18]. Poor CVH score is defined as exhibiting greater than 5 out of these 7 risk factors. This shift represents the AHA’s heightened focus on adverse health behaviors as key contributors to poor CVH outcomes [19].

Following a recent exemplar of a state-level national study of this new metric [20], we examined the relationships between the food and physical activity environment and the new concept of poor CVH score at the county level within a predominantly rural, underserved state. Understanding the relationship between the local environment, social determinants, behaviors, and health outcomes at the county level has significant potential to inform community policy [21] and could help reduce the high burden of obesity and poor CVH in rural states such as Maine [6]. Our goal in this contextual analysis is to inform actionable, community-level interventions that could help improve health outcomes across the state.

## Methods

### Data sources

#### Behavioral Risk Factor Surveillance System

Demographic and individual-level CVH data were obtained from the Behavioral Risk Factor Surveillance System (BRFSS). The BRFSS uses random-digit telephone and cell phone dialing and a multistage sampling design to select a representative sample of non-institutionalized individuals over 18 years of age in all U.S. states and territories [17]. The BRFSS has been widely used in other health behavior studies [22, 23] and has been extensively validated as a reliable survey metric [24]. We aggregated data over 4 years (2011–2014) for statistical stability. Demographic variables included age (years), sex (male or female), race (white or non-white), annual personal income (less than $15,000, $15,000 to $25,000, $25,000 to $50,000, and over $50,000), education (less than high school, high school, some college, graduated college), and self-reported county of residence.

According to the AHA, “poor CVH score” is defined as exhibiting greater than five of the following seven lifestyle behaviors and health factors: current smoking, physical inactivity, obesity, poor diet, hypertension, diabetes, and high cholesterol [19]. To calculate poor CVH score, we first dichotomized each of these self-reported CVH indicators from BRFSS [25]. Our smoking indicator included current smokers who have smoked over 100 cigarettes in their lifetime. Physical inactivity was defined as failure to meet the CDC recommended guidelines of 150 min per week of moderate and/or vigorous activity [26]. Respondents with a body mass index (calculated as self-reported weight divided by self-reported height) of greater than or equal to 30 were categorized as obese. Poor diet was defined as the consumption of less than five daily servings of fruits and vegetables (a reliable estimator of overall diet quality [27]). Respondents reporting high blood pressure, blood sugar, or cholesterol were coded as having hypertension, diabetes, or high cholesterol, respectively.

We then summed the 7 dichotomous metrics for each respondent to acquire a CVH score ranging from 0 to 7, with scores greater than 5 classified as poor CVH [20, 25]. Those with missing, refused, or unknown answers to any of the 7 metrics were excluded (23,876 excluded respondents in total). Since many respondents were excluded from this aggregated score, we also descriptively examined four components of the poor CVH score (poor diet, physical inactivity, obesity, and diabetes) individually to assess specific relationships. We specifically chose to isolate these behaviors and CVH indicators because of their connections to food and physical activity environments and their well-established associations with CVH outcomes [4, 10].

### USDA Economic Research Service Food Environment Atlas

We developed county-level food and physical activity environment variables from data aggregated by the USDA Economic Research Service (ERS) Food Environment Atlas. The Food Environment Atlas is a public database with statistics on over 211 food environment indicators at the county, state, and regional level [7]. This database, which gathers statistics from institutions such as the U.S. Census Bureau and the Supplemental Nutrition and Assistance Program, has been widely used as a reliable estimator of the built environment [12, 16, 20].

We included both county-level “food availability” variables and “food store access” variables in our analysis [16]. The Food Environment Atlas calculated food availability variables based on North American Industry Classification System (NAICS) codes in the 2012 County Business Patterns and U.S. Census Bureau Population Estimates. We included the following food availability variables, measured per 1000 residents: (1) density of “fast-food restaurants”, which include all limited service restaurants in which patrons pay prior to eating (NAICS code 722211); (2) density of “full-service restaurants”, defined as establishments where patrons are seated and pay after eating(NAICS code 722110); (3) density of “grocery stores”, which consist of both supermarkets and small grocery stores (NAICS code 445110); and (4) density of “convenience stores”, which include convenience stores, limited food marts, and gas stations serving food (NAICS code 447110). We also examined the number of “fitness and recreation facilities” per 1000 residents using NAICS code 713940, which encompass both fitness centers (such as the YMCA and community centers with gymnasia) and recreational sport facilities (for instance, skating rinks and indoor tennis courts). For our “food store access” metric, we assessed county percent of households with no car and low store access (classified using GIS analysis as residents living more than 1 mile (urban residents) or 10 miles (rural residents) from a grocery store) [28]. County median household income was also assessed as a county-level wealth indicator.

### Statistical analysis

We first descriptively examined the weighted prevalence of BRFSS health behaviors (poor diet and inactivity) and CVH indicators (obesity, diabetes, and poor CVH score) stratified by demographic and county-level environment characteristics. Maine counties were grouped into low, medium, or high tertiles of each Food Environment Atlas environmental variable [20]. We then assessed the prevalence of BRFSS-reported health behaviors and CVH indicators among the residents of each environment tertile. Prevalence rates were compared across the three tertiles with chi-squared tests of significance.

We then fitted 12 separate multivariable logistic regression models, one for each combination of key CVH indicator of interest (obesity and poor CVH score) and food and physical activity environment variable (6 total). Each model adjusted for demographic characteristics (age, sex, income, and education) and then quantified the relationship between one environment variable (in tertiles) to each CVH indicator. We did not adjust for race due to the very low prevalence of diversity in Maine. Models were not fitted for individual health behaviors (i.e., poor diet and physical inactivity). Modeling environment variables separately with demographic adjustment allowed us to examine the adjusted univariate association of each environment variable with each key CVH indicator. Interaction effects among demographic and environmental variables were assessed and were not found to be statistically significant. For each model, we reported the odds ratio (OR) and 95% confidence interval (CI) associated with predictors. We made the decision not to conduct multi-level models for this analysis, as in this case, we were specifically interested in describing the context (built environment) in relation to each CVH indicator. Multi-level modeling may introduce assumptions that may not be true, and can introduce confounding into models [29, 30]. All analyses were performed using SAS University Edition. This study was reviewed and approved by the Human Subjects Committee at Maine Medical Center.

## Results

We analyzed a total of 40,398 Maine BRFSS respondents in our aggregated 4-year sample, which estimates a weighted total of 4,252,461 adult Maine residents. We found that 79.7% of Maine residents have a poor diet, 44.9% are physically inactive, 28.3% are obese, 9.6% have diabetes, and 17% have poor CVH score. Table 1 demonstrates substantial variation in adverse health behaviors and poor CVH indicators by demographic characteristics. Especially marked differences were noted in the prevalence of overall poor CVH score by income (28.2% among the low personal income group and 10.8% among the high personal income group) and education (32.4% among those with less than a high school education and 8.3% among college graduates).

**Table 1:** Weighted ME Prevalence of Health Behaviors and Poor CVH Across Individual Characteristics, BRFSS 2011-2014^a^

Individual Characteristic	Weighted n	%	Poor diet (%)	Inactive (%)	Obese (%)	Diabetic (%)	Poor CVH (%)
ME state	4,252,461	__	79.7	44.9	28.3	9.6	17.0
Age Group							
18–45	1,695,630	40.1	79.1	44.1	25.2***	2.7***	6.2***
45–64	1,612,818	38.2	79.7	46.1	33.0	11.4	20.3
≥64	918,532	21.7	81.0	44.1	25.9	19.2	25.0
Sex							
Female	2,197,921	51.7	74.8***	45.2	27.8	9.0**	14.8***
Male	2,054,540	48.3	85.0	44.7	28.8	10.3	19.3
Race							
White	3,993,057	95.2	79.8	44.8	28.4	9.5	17
Non-White	202,050	4.8	78.1	47.3	27.7	9.7	17.9
Personal Income							
<$15,000	450,109	11.9	83.3***	51.1***	33.7***	15.4***	28.2***
$15,000 to <$25,000	702,047	18.6	83.9	51.2	32.9	13.7	24.9
$25,000 to <$50,000	1,082,144	28.6	81.7	45.7	30.0	9.7	17.7
≥$50,000	1,548,240	40.9	75.3	39.0	25.0	6.2	10.8
Education							
Less than high school	424,504	10.0	85.1	53.9	33.0	14.9	32.4
High school or equivalent	1,464,257	34.5	84.8	51.4	31.4	11.2	21.4
Some college	1,290,851	30.5	79.1	43.9	29.2	9.0	15.8
Graduated college	1,059,278	25.0	71.4	33.8	21.1	6.1	8.3

Results based on 40,398 respondents surveyed in the Maine BRFSS from 2011 to 2014. Percentages reflect BRFSS weighted estimates for Maine

Chi-squared statistical significance within category (*p < 0.05; **p < 0.01; ***p < 0.0001)

Abbreviations: BRFSS, Behavioral Risk Factor Surveillance System; CVH, cardiovascular health; ME, Maine

^a^Number of missing responses by category (): Age Group (317); Sex (0); Race (606); Income (4405); Education (146); Poor diet (20,359); Inactive (20,941); Obese (1747); Diabetic (45); Poor CVH (23,876)

Table 2 shows the distribution of adverse health behaviors and poor CVH indicators by county-level environment characteristics (see Additional file1 for detailed individual county characteristics). A greater proportion of individuals living in counties with high densities of fast food restaurants and full-service restaurants had poor diet, were obese, and had poor CVH. We also found strong positive associations between convenience store density and poor CVH indicators. Compared to residents in counties with high fitness facility density, a greater proportion of those living in areas with fewer fitness facilities were physically inactive (49.1% vs. 42.3%), obese (31.5% vs. 26.0%), and had poor CVH score (20.9% vs. 14.9%) (all *p* < 0.0001).

**Table 2:** Weighted Prevalence of CVH Indicators Across Maine County-Level Built Environment Characteristics, BRFSS 2011–2014 and USDA Atlas 2010–2012^a^

County-Level Characteristic	County Tertile^b^	Tertile Minimums^b^	Weighted n	Percent	Poor Diet (%)	Physically Inactive (%)	Obese (%)	Diabetic (%)	Poor CVH (%)
Fast Food Restaurants (per 1000)	Low	0	1,331,590	32.6	82.1	47.8	30.6	10.4*	19.8***
	Med	0.59	1,582,067	38.7	79.0	44.4	29.3	9.6	16.8
	High	0.75	1,175,944	28.8	78.1	42.1	25.3	9.0	14.8
Full-Service Restaurants (per 1000)	Low	0	1,532,443	37.5	81.8	47.4	31.7	10.5***	19.2***
	Med	0.85	1,218,700	29.8	80.5	45.6	29.5	10.4	18.5
	High	1.29	1,338,458	32.7	76.7	41.3	24.1	8.1	13.8
Grocery Stores (per 1000)	Low	0	1,167,586	28.6	81.5	47.0	31.5	10.4**	18.7***
	Med	0.21	1,985,350	48.5	78.3	42.6	26.5	8.9	15.3
	High	0.28	936,664	22.9	80.5	47.0	29.3	10.4	19.3
Convenience Stores (per 1000)	Low	0	1,498,964	36.7	77.*	40.7	24.7	8.5***	14.0***
	Med	0.64	1,659,982	40.6	80.8	46.4	30.7	10.1	18.0
	High	0.78	930,654	22.8	82.1	48.9	30.8	11	20.9
Fitness Facilities (per 1000)	Low	0	1,393,978	34.1	82.9	49.1	31.5	10.9***	20.9***
	Med	.08	1,142,328	27.9	78.5	43.4	28.4	9.2	15.9
	High	.11	1,553,293	38.0	77.8	42.3	26.0	9.0	14.9
Poor Access To Store and No Car (%)	Low	0	1,017,409	24.9	79.1	44.7	29.1	9.8**	17.1***
	Med	2.04	2,079,755	50.9	78.8	43.2	26.7	9.1	15.5
	High	2.95	992,435	24.3	82.3	48.5	31.8	10.9	20.7
County Median House hold Income ($)	Low	0	1,007,074	24.6	83.2	49.0	32.5	11.3***	21.5***
	Med	41,106	1,469,936	35.9	80.4	46.1	30.1	10.0	17.6
	High	45,520	1,612,589	39.4	77.0	41.1	24.6	8.5	14.2

Results based on 40,398 respondents surveyed in the Maine BRFSS from 2011 to 2014. Percentages reflect BRFSS weighted estimates for Maine

Chi-squared statistical significance within category (*p < 0.05; **p < 0.01; ***p < 0.0001)

Abbreviations: BRFSS, Behavioral Risk Factor Surveillance System; CVH, cardiovascular health; USDA, United States Department of Agriculture

^a^Number of missing responses by category (): All County-Level Characteristics (1195); Poor diet (20,359); Inactive (20,941); Obese (1747); Diabetic (45); Poor CVH (23,876)

^b^Tertile cutoffs are reported in the units corresponding to each county built environment variable. The ‘low’ tertile includes counties in the lowest third of each built environment variable, the ‘med’ tertile includes counties in the middle third, and the ‘high’ tertile includes counties in the highest third

**Fig. 1:**
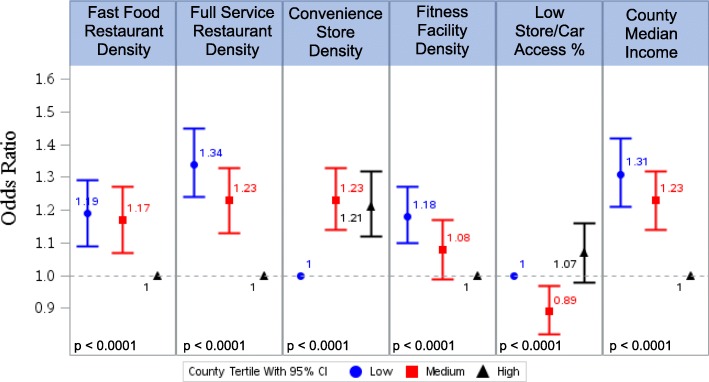
Relationships Between Built Environment Characteristics and Obesity: Results From Six Multivariable Logistic Regression Models Each of the six models includes demographic variables (age, sex, personal income, education) as adjustors The ‘low’ tertile includes counties in the lowest third of each built environment variable, the ‘med’ tertile includes counties in the middle third, and the ‘high’ tertile includes counties in the highest third The reference tertile for each model is indicated by an odds ratio equal to 1

Figure 1 highlights the relationship between built environment characteristics and obesity after taking demographic factors into account (see Additional file 2 for detailed results from the multivariable models). After adjustment for demographic controls (age, sex, personal income, and education), environmental characteristics most strongly positively associated with obesity included low density of full service restaurants (OR 1.34; 95% CI 1.24–1.45) and low county median household income (OR 1.31; 95% CI 1.21–1.42) as compared with each high group. Conversely, living in an area with a high density of convenience stores was associated with increased odds of obesity compared to living in a county with a low density of convenience stores (OR 1.21; 95% CI 1.12–1.32).

**Fig. 2:**
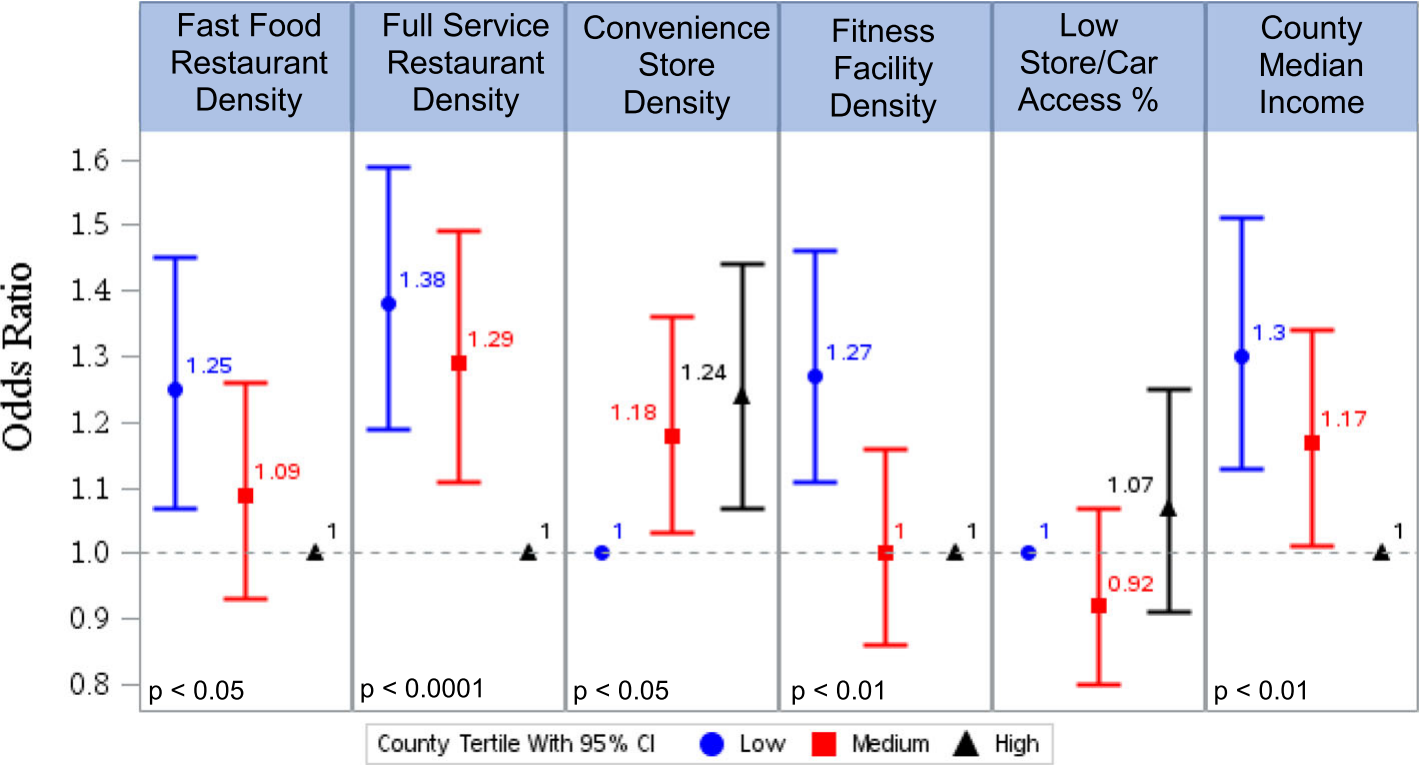
Relationship Between Built Environment Characteristics and Poor CVH: Results From Six Multivariable Logistic Regression Models Each of the six models includes demographic variables (age, sex, personal income, education) as adjustors The ‘low’ tertile includes counties in the lowest third of each built environment variable, the ‘med’ tertile includes counties in the middle third, and the ‘high’ tertile includes counties in the highest third The reference tertile for each model is indicated by an odds ratio equal to 1

As shown in Fig. 2, we observe significant positive associations between aspects of the built environment and overall poor CVH score (see Additional file 2 for detailed results from the multivariable models). The strongest county-level environment predictors of poor CVH were low county median household income (OR 1.30; 95% CI 1.13–1.51), low density of full-service restaurants (OR 1.38; 95% CI 1.19–1.59), and low density of fitness facilities (OR 1.27; 95% CI (1.11–1.46) as compared with the highest density county for each built environment characteristic. Grocery store prevalence and prevalence of households with poor access to the store and no car were not associated with obesity or poor CVH score.

## Discussion

We report strong associations between the built environment and overall poor CVH at the county level within a rural state. After adjustment for personal income and education, food and physical activity environment characteristics such as low density of full-service restaurants, low access to fitness facilities, and high density of convenience stores remain associated with obesity and poor CVH score. Additionally, even after accounting for personal income, low county median income was associated with increased odds of obesity and poor CVH score.

While others have assessed the impact of the built environment on either health behaviors [12, 21] or health outcomes [9, 31], this study confirms and extends this body of work by highlighting how food and physical activity environments may be linked to new, more comprehensive metrics of overall CVH status. Given the disproportionate burden of health disparities and cardiovascular deaths in rural areas [5], we aimed to build upon recent work that examines the relationship between the built environment and health outcomes in these underserved communities [16, 32]. Since population-level characteristics vary significantly by county in this state [33], we highlight the impact of the local built environment after adjusting for demographic covariates. As other exemplary studies have demonstrated, small-area findings such as these are important because they are actionable at the community level [32, 34]. We present county-level environment and demographic relationships within a rural state that may have the potential to inform community planning and policy [21].

Consistent with abundant prior findings, we demonstrate that demographic factors are strong predictors of overall poor CVH [35] and obesity [36]. In accordance with the AHA [37], we found that less education and low personal income are strongly associated with poor diet and physical inactivity, obesity, diabetes, and poor CVH score. This consistent relationship across health behaviors, CVH score, and obesity supports prior research demonstrating relationships between socioeconomic status and adverse health behaviors that contribute to health outcomes [36].

We also demonstrate important relationships between the underlying environment and CVH indicators. For instance, even after adjustment for personal reported income, low county median income predicts both obesity and poor CVH score at the county level. That is, overall poverty of an area may affect health regardless of one’s individual income. One explanation for this environmental impact of overall poverty could be the lack of access to comprehensive medical care in rural, impoverished areas of Maine [38]. Low county median income may be linked to limited health services and thus poorer outcomes across all levels of personal income. This relationship highlights the potential influence of the underlying environment in predicting CVH indicators.

Food environment metrics exhibit varied associations with obesity and CVH indicators [20, 39]. Low county-level density of full-service restaurants is positively associated with both obesity and poor CVH score (specifically, poor diet and physical inactivity), which is consistent with several prior studies [16, 40]. Unexpectedly, living in a county with fewer fast-food restaurants is also associated with poorer health behaviors and outcomes. As Ahern and colleagues [16] suggest, the relationship between fast food restaurants and health may be complex in rural areas; for instance, fast food restaurants along interstates near outdoor recreation areas in Maine may cater to tourists rather than local residents. Others have argued that proximity to fast food restaurants may not be associated with actual fast-food consumption [41, 42]. Future longitudinal studies of the association between fast food restaurants and CVH indicators may provide further evidence in support of this unexpected finding. Grocery store density has no directional association with adverse health behaviors and CVH outcomes, which may be indicative of the range of both healthful and unhealthful (e.g. packaged) foods at these establishments [20]. Consistent with others [11, 16], we found that living in a county with a high density of convenience stores (vs. low density) is associated with poor diet, obesity, and poor CVH score. This may reflect the limited availability of fresh fruit and vegetables and unprocessed foods available at these establishments [43, 44].

As expected, low Maine county density of recreation facilities is strongly associated with both obesity and poor CVH score (physical inactivity in particular). Many other studies have demonstrated the positive relationship between availability of recreation facilities and improved health outcomes [14]; however, only a few have examined this metric within a rural state [45]. This finding accentuates the importance of implementing recreation facilities and exercise opportunities within rural areas.

### Implications

This research demonstrates the potential impact of community-level population health interventions in a rural state. With the highest obesity rate in New England and a significant burden of cardiovascular disease, Maine has a marked need for actionable community-specific interventions that target these health issues. As this study suggests, the local food and physical activity environment may be one avenue for community interventions. This could include enhanced access to recreational facilities, such as free indoor walking programs in public school gymnasia, and efforts to improve access to fruits, vegetables, and unprocessed foods in small grocery establishments. These environmental improvements may be accompanied by educational initiatives, policy measures, and media campaigns that further encourage behavior change [21]. Understanding the local built activity environment will strengthen population health efforts to reduce the burden of obesity and cardiovascular disease.

### Limitations

Our study has several limitations. Data on health behaviors and health conditions were limited to self-reported responses in the 2011–2014 BRFSS; however, this survey has been validated as an accurate measure of health behaviors and conditions [24]. Since Maine is a state with minimal racial and ethnic diversity, we were limited in our ability to examine differences in that regard. Due to the nature of the data use agreement, this study presents data from the 2011–2014 BRFSS, and does not reflect changes in health behaviors, health conditions, and demographics in the years since then.

Our metrics also cannot capture the entire breadth of the food and physical activity environment. For example, our physical activity environment variable does not include outdoor spaces such as parks, hiking trails, and walking paths, which are common activity venues in rural states that are also associated with lower rates of obesity and better cardiovascular outcomes [15]. We were also unable to capture environmental variation within counties (e.g., variation across individual towns) since data presented in the USDA Food Environment Atlas were only available at the county level (see Additional file 1 for more information about variation across counties). While the U.S. Census Bureau is a well-regarded source of geographical data, there may be gaps or miss-classifications of built environment establishments, especially in rural Maine where data can be difficult to verify. Finally, this study presents 2010–2012 metrics of the food and physical activity environment. Further research is needed in order to investigate how the relationship between the built environment and health outcomes has changed in recent years.

Although demographic and environmental factors are widely regarded as strong contributors to health, there are many other factors that influence outcomes that we could not consider (e.g., health care access, drug use, genetics). Additionally, environmental factors are often correlated; while we focused on isolating the impact of each individual environment factor on health outcomes in separate models, we hope that future studies build upon this to explore how the combined context of multiple complex environmental components and potential neighborhood clustering may influence health outcomes. Since our study is cross-sectional, it is impossible to draw causal inferences from our findings. However, studies of relationships between environment and the health of communities are important as we strive to improve population health.

## Conclusions

We demonstrate that, in a poorer rural state, there are measurable relationships between food and physical activity environments and important health outcomes, even after adjustment for income and education. As is widely known, social determinants of health are critical drivers of health outcomes [32, 37]. However, this study points to additional relationships between certain aspects of food and physical activity environments and self-reported health. Changes to community built environments may represent considerable opportunities for population health improvement initiatives. These findings thus help inform communities and policymakers of the contextual factors that influence health behaviors and outcomes.

## Additional files

Additional file 1: Maine County Characteristics and Weighted Prevalence of Health Behaviors and Poor CVH, BRFSS 2011–2014, USDA Atlas 2010–2012 and 2010 U.S. Census. (DOCX 21 kb)

Additional file 2: Relationships Between Built Environment Characteristics and Obesity and Poor CVH: Detailed Results and Confidence Intervals From Six Multivariable Logistic Regression Models. (DOCX 19 kb)

## Abbreviations

AHA: American Heart Association
BMI: Body mass index
BRFSS: Behavioral Risk Factor Surveillance System
CDC: Center for Disease Control
CVH: Cardiovascular health
ERS: Economic Research Service
ME: Maine
NAICS: North American Industry Classification System
USDA: U.S. Department of Agriculture
