# Supplementary material for: Association between the food and physical activity environment, obesity, and cardiovascular health across Maine counties
Source: BMC Public Health. 2019 Apr 3;19:374. doi: 10.1186/s12889-019-6684-6 (PMC6448221; doi:10.1186/s12889-019-6684-6)
Supplement: Supplementary file 1 — Maine County Characteristics and Weighted Prevalence of Health Behaviors and Poor CVH, BRFSS 2011–2014, USDA Atlas 2010–2012 and 2010 U.S. Census. (DOCX 21 kb) [file 12889_2019_6684_MOESM1_ESM.docx]

Additional File 1. Maine County Characteristics and Weighted Prevalence of Health Behaviors and Poor CVH, BRFSS 2011-2014, USDA Atlas 2010-2012 and 2010 U.S. Census.

| **County** | **Land area (square miles)** | **Population over 18 (n)** | **BRFSS**  **n** | **Weighted n** | **Poor Diet (%)** | **Physically Inactive (%)** | **Obese (%)** | **Diabetic (%)** | **Poor CVH (%)** |
| --- | --- | --- | --- | --- | --- | --- | --- | --- | --- |
| Androscoggin | 467.9 | 83,394 | 2,703 | 326,113 | 81.9 | 27.4 | 34.5 | 11.4 | 20.1 |
| Aroostook | 6,671.3 | 57,486 | 2,135 | 236,815 | 85.0 | 30.7 | 35.0 | 13.8 | 24.7 |
| Cumberland | 835.2 | 222,780 | 6,515 | 814,892 | 76.3 | 20.2 | 22.9 | 7.8 | 12.2 |
| Franklin | 1,696.6 | 24,721 | 1,478 | 107,590 | 82.8 | 27.9 | 31.7 | 9.9 | 23.6 |
| Hancock | 1,586.9 | 44,441 | 1,791 | 178,113 | 76.0 | 25.0 | 26.6 | 8.2 | 14.4 |
| Kennebec | 867.5 | 96,843 | 3,437 | 386,543 | 79.9 | 25.9 | 30.8 | 9.8 | 17.0 |
| Knox | 365.1 | 32,026 | 1,938 | 124,237 | 76.1 | 23.2 | 22.4 | 7.9 | 12.2 |
| Lincoln | 455.8 | 27,989 | 1,826 | 113,625 | 75.6 | 28.3 | 23.4 | 8.7 | 16.1 |
| Oxford | 2,076.8 | 45,516 | 1,722 | 192,753 | 80.9 | 29.8 | 30.8 | 10.3 | 19.0 |
| Penobscot | 3,397.3 | 123,568 | 3,728 | 454,930 | 82.8 | 29.6 | 29.9 | 10.3 | 19.3 |
| Piscataquis | 3,960.9 | 14,170 | 921 | 68,026 | 85.1 | 30.9 | 34.8 | 12.2 | 19.5 |
| Sagadahoc | 253.7 | 27,871 | 1,450 | 106,400 | 76.1 | 21.7 | 25.2 | 9.6 | 18.0 |
| Somerset | 3,924.4 | 41,052 | 1,456 | 172,103 | 84.5 | 37.5 | 34.6 | 11.4 | 22.8 |
| Waldo | 729.9 | 30,639 | 1,751 | 121,530 | 80.5 | 28.0 | 29.5 | 9.3 | 15.5 |
| Washington | 2,562.7 | 26,292 | 1,749 | 108,257 | 82.7 | 31.0 | 29.8 | 10.6 | 22.4 |
| York | 990.7 | 155,040 | 4,603 | 577,672 | 78.4 | 25.8 | 27.3 | 9.1 | 15.7 |

*Note:* Results based on 40,398 respondents surveyed in the Maine BRFSS from 2011-2014. Percentages and reflect BRFSS weighted estimates for Maine adult population. Adult population extracted from the 2010 U.S. Census Population Estimates.

Abbreviations: BRFSS, Behavioral Risk Factor Surveillance System; CVH, cardiovascular health; USDA, United States Department of Agriculture

^a^Number of missing responses by category (): County (1,095); Poor diet (20,359); Inactive (20,941); Obese (1,747); Diabetic (45); Poor CVH (23,876)
