# Supplementary material for: Association between the food and physical activity environment, obesity, and cardiovascular health across Maine counties
Source: BMC Public Health. 2019 Apr 3;19:374. doi: 10.1186/s12889-019-6684-6 (PMC6448221; doi:10.1186/s12889-019-6684-6)
Supplement: Supplementary file 2 — Relationships Between Built Environment Characteristics and Obesity and Poor CVH: Detailed Results and Confidence Intervals From Six Multivariable Logistic Regression Models. (DOCX 19 kb) [file 12889_2019_6684_MOESM2_ESM.docx]

Additional File 2. Relationships Between Built Environment Characteristics and Obesity and Poor CVH: Detailed Results and

Confidence Intervals From Six Multivariable Logistic Regression Models^a^

| **Variable** | **Level^b^** | **Obesity OR (CI)^c^** | **Poor CVH OR (CI)^c^** |
| --- | --- | --- | --- |
| **Fast food restaurants**  **(per 1,000)** | Low | 1.19 (1.09-1.29) | 1.25 (1.07-1.45) |
|  | Med | 1.17 (1.07-1.27) | 1.09 (0.93-1.26) |
|  | High | 1.00 (Reference)*** | 1.00 (Reference)* |
| **Full service restaurants (per 1,000)** | Low | 1.34 (1.24-1.45) | 1.38 (1.19-1.59) |
|  | Med | 1.23 (1.13-1.33) | 1.29 (1.11-1.49) |
|  | High | 1.00 (Reference)*** | 1.00 (Reference)*** |
| **Convenience stores**  **(per 1,000)** | Low | 1.00 (Reference)*** | 1.00 (Reference)* |
|  | Med | 1.23 (1.14-1.33) | 1.18 (1.03-1.36) |
|  | High | 1.21 (1.12-1.32) | 1.24 (1.07-1.44) |
| **Fitness facilities**  **(per 1,000)** | Low | 1.18 (1.10-1.27) | 1.27 (1.11-1.46) |
|  | Med | 1.08 (0.99-1.17) | 1.00 (0.86-1.16) |
|  | High | 1.00 (Reference)*** | 1.00 (Reference)** |
| **Poor access to store and no car (%)** | Low | 1.00 (Reference)*** | 1.00 (Reference) |
|  | Med | 0.89 (0.82-0.97) | 0.92 (0.80-1.07) |
|  | High | 1.07 (0.98-1.16) | 1.07 (0.91-1.25) |
| **County median income ($)** | Low | 1.31 (1.21-1.42) | 1.30 (1.13-1.51) |
|  | Med | 1.23 (1.14-1.32) | 1.17 (1.01-1.34) |
|  | High | 1.00 (Reference)*** | 1.00 (Reference)** |

P value for odds ratios (*p<0.05; **p<0.01; ***p<0.0001).

Abbreviations: CI, confidence interval; CVH, cardiovascular health; OR, odds ratio.

^a^Each of the six models includes demographic variables (age, sex, personal income, education) as adjustors.

^b^The ‘low’ tertile includes counties in the lowest third of each built environment variable, the ‘med’ tertile includes counties in the middle third, and the ‘high’ tertile includes counties in the highest third.

^c^The reference tertile for each model is indicated by an odds ratio equal to 1.
